# Examining Behavioural Coping Strategies as Mediators between Work-Family Conflict and Psychological Distress

**DOI:** 10.1155/2015/343075

**Published:** 2015-01-28

**Authors:** Sanaz Aazami, Khadijah Shamsuddin, Syaqirah Akmal

**Affiliations:** ^1^Faculty of Nursing and Midwifery, Ilam University of Medical Science, Ilam 56000, Iran; ^2^Department of Community Health, Faculty of Medicine, Universiti Kebangsaan Malaysia, 56000 Cheras, Kuala Lumpur, Malaysia

## Abstract

We examined the mediating role of behavioral coping strategies in the association between work-family conflict and psychological distress. In particular, we examined the two directions of work-family conflict, namely, work interference into family and family interference into work. Furthermore, two coping styles in this study were adaptive and maladaptive coping strategies. This cross-sectional study was conducted among 429 Malaysian working women using self-reported data. The results of mediational analysis in the present study showed that adaptive coping strategy does not significantly mediate the effect of work-family conflict on psychological distress. However, maladaptive coping strategies significantly mediate the effect of work-family conflict on psychological distress. These results show that adaptive coping strategies, which aimed to improve the stressful situation, are not effective in managing stressor such as work-family conflict. We found that experiencing interrole conflict steers employees toward frequent use of maladaptive coping strategies which in turn lead to psychological distress. Interventions targeted at improvement of coping skills which are according to individual's needs and expectation may help working women to balance work and family demands. The important issue is to keep in mind that effective coping strategies are to control the situations not to eliminate work-family conflict.

## 1. Introduction

The psychologically important aspects of everybody's life are employment, marriage, and parenting [1]. Engaging in work, family, and parenting roles could have a constructive impact on an individual's life [2]. Nevertheless, lack of ability to balance the responsibilities associated with these roles can lead to conflict between work and family domains [3].

Work-family conflict is frequently defined as “a form of interrole conflict” in which the behavioural requirements associated with the role performed in the work and family domains are mutually incompatible [4]. Initially, it is supposed to be a unidimensional construct but Gutek et al. [5] proposed a bidirectional nature of conflict between work and family. Family roles and responsibilities interfere into work role, which is called Family Interference into Work (FIW). On the other hand, work roles and responsibilities can interfere with family role, which is called Work Interference into Family (WIF). This is a reciprocal relationship; in which work issues occur if the job obligations remain unfulfilled because a person's family interferes with work. Consequently, work issues spill over into the family matters, causing work to interfere with family domain. Therefore, the two directions of work-family interference need to be distinguished and assessed separately because each one may have a unique set of consequences. In this study, we used the two directions of WIF and FIW to examine their outcomes separately.

Conflicting demands between work and family is shown to be associated with organizational consequences, such as turnover intention [6], job dissatisfaction [7], absenteeism, lower job commitment [8], and burnout [9]. On the other hand, experiencing work-family conflict especially among employed women has become a great source of stress [10] which can affect individuals' health and wellbeing [11]. This can be explained by lower level of stress among individuals who have a balanced work-family life. Those individuals who balanced their work and family demands, use routines that avoid long term and chronic work-family conflict. In addition, balanced individuals perform their role's demands in low stressful situations possibly due to the fact that they are enacting in their salient role [11]. In general, a balanced involvement in work and family roles reduces the level of role interference and decreases the level of stress that eventually promotes health and wellbeing of employees.

Several workplace policies have been recommended to minimize the effect of work on family life and they included a variety of programs, such as maternal and paternal leave, childcare facilities, and flexible work arrangement. However, such programs and policies are still not being offered to everyone. Furthermore, the functional capabilities of individuals at work might be affected by the way they cope with the concurrent demands of job and family. Coping strategies are of critical importance to attenuate the stress when a woman experiences conflict between work and household responsibilities [12].

Exploring the nature of coping strategies is not a new concept, and there has been a long history of theories that tried to distinguish different styles of coping strategies [13, 14]. Three most common classifications for coping strategies include emotion-focused versus problem-focused coping, approach versus avoidance, and, lastly, cognitive versus behavioural (see [15] for a review). However, Skinner et al. [15] argues that it is better to categorize coping strategies based on their adaptation process. That means, a specific way of coping can be considered as adaptive if, it is chosen to handle stressful situations and eventually reduces physical and psychological burdens of the stress. In general, Skinner et al. [15] believes that every possible way of coping can be considered as adaptive or maladaptive based on three main criteria, including (1) long-term consequences, (2) their subjective experience, and, finally, (3) their present qualities.

Work-family conflict is considered as a work-stress, which can adversely affect mental health status of the employees [16]. However, little attention has been paid to the mediating role of behavioural coping strategies in the effect of work-family conflict on psychological wellbeing. Therefore, the aim of this study is to examine the mediating role of behavioural coping strategies (adaptive/maladaptive approaches) on the association between interrole conflict (WIF/FIW) and psychological distress.

## 2. Methods

This cross-sectional study was a part of a broader study conducted in Malaysia during seven months of data collection from August 2012 to February 2013. This study was conducted among 429 Malaysian women who were working in the public service departments of Kuala Lumpur and Selangor. Multistage simple random sampling method was used to recruit respondents. Participants were informed about their voluntary right to participate in this study and a written consent was obtained prior to actual data collection. Malaysian women who were married and worked for at least 6 months in the respective departments were included in the study. However, women who had a history of mental illness and were under treatment of psychiatric drugs were not eligible to be included in this study. We used a series of self-reported questionnaires, including sociodemographic backgrounds, work-family conflict questionnaire, and brief-COPE inventory.

## 3. Instruments

### 3.1. Sociodemographic Profile

Respondents were asked to identify their age, number of children, years of working experience, working hour per week, and their level of education. These variables were used as control variables in further analysis.

### 3.2. Work-Family Conflict Questionnaire

Work-family conflict questionnaire was developed by Kelloway et al. [17] to measure interrole conflict. This questionnaire consisted of 22 items with Likert-type scale with answers ranging from 1 = strongly disagree to 5 = strongly agree. This questionnaire is able to measure the two directions of work-family conflict, namely, Work Interference into Family (WIF) and Family Interference into Work (FIW). The score of each direction ranged from 11 to 55 and higher score indicated a higher level of perceived work-family conflict. The Malay version of this questionnaire had been previously validated [18] among Malaysian working women. Therefore, we used the validated Malay version of the work-family conflict questionnaire.

### 3.3. Brief COPE Inventory

The brief COPE scale was developed by Carver [19] to examine a comprehensive feature of behavioural coping strategies among adults. The 28 items COPE is rated by three points Likert-type scale ranging from 0 (I have not been doing this at all) to 3 (I have been doing this a lot). The brief COPE scale showed acceptable results of internal structure and reliability analysis [19]. In order to classify the coping strategies into further groups, Carver et al. [20] recommended to conduct a second-order factor analysis. Meanwhile, every sample has its own unique characteristics and categorizing several ways of coping into adaptive and maladaptive approaches should be based on the nature of a specific stress. Therefore, we reclassified the fourteen coping strategies using second-order factor analysis (e.g., adaptive and maladaptive approaches). The brief COPE scale had been validated in Malay language among breast cancer patients [21]. In Yusoff's et al. [21] study, brief-COPE scale had been categorised into 14 subscales with reported internal consistency (*α* Cronbach) ranged from 0.51 to 0.99.

## 4. Statistical Analysis

The analyses in this study began with the description of sample characteristics, as well as the mean and standard deviation of work-family conflict, psychological distress, and, finally, the fourteen coping strategies. In order to identify subgroups of the coping strategies, second-order factor analysis was conducted, which extracted two broader dimensions of coping strategies. The two dimensions were named adaptive coping and maladaptive coping. The intercorrelations of study variables were tested using binary correlation analysis. Finally, Baron and Kenny's [22] steps for testing mediation paths were used to assess the indirect effect of work-family conflict on psychological distress via the role of behavioural coping strategies. Baron and Kenny's test included three steps. Firstly, independent variables (the two dimensions of work-family conflict) should be significantly related to psychological distress. Secondly, the two dimensions of work-family conflict should be significantly related to the mediators (adaptive and maladaptive coping strategies). Thirdly, the mediators should be significantly related to psychological distress in the presence of two dimensions of work-family conflict. If all three conditions were met, partial mediation is established. Full mediation is established if there is nonsignificant beta for independent variables in the model that included psychological distress, work-family conflict and coping strategies.

## 5. Results

The study sample consisted of 429 married working women aged 34.7 ± 8.9 years with 1.7 ± 1.5 children. Women in this study had been working for 10.6 ± 9.3 years on average with the mean 42.6 ± 5.2 working hours per week. Regarding educational achievement, only 4.4% (19) of respondents in this study had obtained a postgraduate degree, 33.3% (143) had bachelor degree, 34.3% (147) had diploma, and 28% (120) were educated at secondary school level. However, for further analysis, education was dichotomized and coded as 0 = no university degree and 1 = university degree.

Table 1 shows the mean score of fourteen behavioural coping strategies frequently used by women in this study. The most frequent strategy used was religion (4.77 ± 1.42), followed by active coping (4.11 ± 1.42), planning (4.09 ± 1.36), positive reframing (3.98 ± 1.49), acceptance (3.96 ± 1.53), instrumental support (3.75 ± 1.57), self-distraction (3.75 ± 1.53), emotion (3.35 ± 1.67), venting (2.61 ± 1.42), humour (2.14 ± 1.75), self-blame (2.04 ± 1.47), denial (0.99 ± 1.32), behavioural disengagement (0.99 ± 1.32), and, lastly, substance use (0.14 ± 0.65). In the next step, we run the second-order factor analysis to identify subgroups of behavioural strategies that were used to cope with the conflicting demands of work and family.

Principal Component Analysis (PCA) with Direct Oblimin Rotation was conducted and items were restricted to factor loading of more than 0.40. Two dimensions were extracted with an eigenvalue of more than 1, which accounted for 54.86% of the variance. After observing behavioural strategies, the dimensions were named adaptive coping (first dimension) and maladaptive coping (second dimension) strategies. All of the items were loaded in one dimension only.

Adaptive coping strategy accounted for 37.3% of the variance and was a broader dimension of several strategies. These strategies included active coping, positive reframing, acceptance, planning, use of instrumental support, use of emotional support, religion, self-distraction, venting, and humour. The second dimension (maladaptive coping) explained 17.55% of the variance and included a variety of strategies, namely, denial, behavioural disengagement, substance use, and self-blame. The value of Cronbach's alpha was 0.88 for adaptive coping and 0.75 for maladaptive coping.

Baron and Kenny's steps were used to test the mediating role of coping strategies on the association between work-family conflict and psychological distress. The following assumptions need to be established before testing the mediated model.

1. Is there a significant association between the two work-family conflict dimensions (WIF and FIW) and psychological distress?
2. Is there a significant association between the two work-family conflict dimensions (WIF and FIW) and adaptive and maladaptive coping strategies?

Table 2 shows that the first prerequirement had been established since both WIF (*r* = 0.35, *P* < 0.01) and FIW (*r* = 0.45, *P* < 0.01) were significantly correlated with psychological distress. However, adaptive coping strategy was significantly associated with only WIF (*r* = 0.10, *P* < 0.05) but not FIW. On the other hand, maladaptive coping strategy was significantly associated with both WIF (*r* = 0.16, *P* < 0.01) and FIW (*r* = 0.22, *P* < 0.01). Therefore, our study is capable of examining three mediational models: (a) whether adaptive coping strategies mediate the association between WIF and psychological distress, (b) whether maladaptive coping strategies mediate the association between WIF and psychological distress, and (c) whether maladaptive coping strategies mediate the association between FIW and psychological distress.

In order to examine the three mediation models, we run hierarchical linear regression (Table 3). Psychological distress regressed on WIF and FIW in the presence of adaptive and maladaptive coping strategies. Each model contained three steps in which control variables (work hour, working experience, number of children, and education) were included in the first step, followed by antecedent (WIF or FIW) and, finally, mediators (adaptive or maladaptive coping). Model (a) examined the mediating role of adaptive coping strategy on the association between WIF and psychological distress. However, this model is not significant since the adaptive coping strategy (*b* = −0.02, *P* = 0.65) did not significantly predict psychological distress. Therefore, our results showed that employing adaptive coping strategies did not minimize the effect of WIF on psychological distress.

Regarding model (b), which examined the mediating role of maladaptive coping strategies on the association between WIF and psychological distress, our results showed a significant mediational path. Employing maladaptive coping strategies (*b* = 0.33, *P* < 0.001) had significantly increased psychological distress. On the other hand, in the presence of maladaptive coping strategies, the effect of WIF on psychological distress still is significant but beta weight at the second step (*b* = 0.30, *P* < 0.01) is reduced (*b* = 0.36, *P* < 0.01) in the third step. The reduction in beta weight of WIF typically showed the mediating effect of maladaptive coping strategies in the linkage between WIF and psychological distress.

The last model (c) assessed the mediating role of maladaptive coping strategies in the association between FIW and psychological distress. This model showed that maladaptive coping strategies (*b* = 0.33, *P* < 0.001) had significantly predicted higher psychological distress. Furthermore, WIF (*b* = 0.33, *P* < 0.001) directly predicted psychological distress (in the second step) and after introducing the mediator into the model, WIF (*b* = 0.26, *P* < 0.001) was still significant but the beta weight was reduced. The reduction of beta weight in FIW shows that employing maladaptive coping strategies had significantly worsened the adverse effect of FIW on psychological distress.

## 6. Discussion

This study sought to examine the potential mediating role of coping strategies in the effect of work-family conflict on psychological distress. In particular, we aimed to examine the two directions of work-family conflict, namely, WIF and FIW, as well as two coping strategies (adaptive and maladaptive coping).

Our results showed that coping strategies among Malaysian working women can be categorized into two broader dimensions. The first dimension, which is recognised as “adaptive coping,” was a combination of broader coping strategies including active coping, positive reframing, acceptance, planning, use of instrumental support, use of emotional support, religion, self-distraction, venting, and humour. Besides that, the second dimension is recognized as “maladaptive coping” and included four behavioural strategies, namely, denial, behavioural disengagement, substance use, and self-blame.

This study showed that the adaptive coping strategy is significantly correlated with WIF, but not FIW. In other words, women who experience a high level of work-family conflict tried to manage the situation by adaptive coping strategies. However, there was no association between FIW and use of adaptive coping strategies. This difference could be attributed to the nature of the family role, which is less rigid in structure. Individuals can easily reschedule family events (e.g., family meals) without any serious consequences. In contrast, the worker role is more structured and more constrained. There is a common norm which expects employees to keep their family lives out of the workplace, while it is not rare to talk about work-related events at family gatherings [17]. Additionally, it had been shown that individuals tend to use problem-focused coping strategies when they feel capable of influencing and controlling the stress [14, 23].

Pertaining to the association between interrole conflict and maladaptive coping, our results showed that there was a significant positive correlation between both WIF and FIW with maladaptive coping strategies. These findings showed that conflicting demands between work and family had led the employees towards frequent usage of maladaptive coping strategies. This finding could be related to the ability of such strategies to act as a regulator of emotions evoked by stress, which at least has stress-reducing properties [24]. However, adapting this type of coping strategies changes nothing and the stressful situations exist as they were. Consequently, failure to manage the problem may lead to the feeling that the situation is being out of control.

Findings from mediation analysis showed that the adaptive coping strategies did not mediate the effect of WIF and FIW on psychological distress. This finding added new knowledge to the field of coping with work-family conflict and mental health status of working women. Adaptive coping strategies logically are defined as strategies of trying to solve problems and managing the situation, which in turn lead to constructive and healthy outcomes. However, we found that in stressful situations, such as work-family conflict, this kind of coping style is not effective in reducing mental health outcomes. A possible explanation for this finding could be the inability of adaptive coping strategies in managing uncontrolled situations of work and family demands [25]. However, our findings are not able to make any conclusion about the long-term effect of adaptive coping strategies on mental health status. This goal can be obtained by future longitudinal research.

This study showed that experiencing work-family conflict led to higher levels of psychological distress. According to the role salient theory [26], women who perceive their valued domain is being threatened are more likely to suffer from poor psychological wellbeing. Furthermore, our mediation analyses revealed that the maladaptive coping strategies had significantly worsened the adverse effect of WIF and FIW on psychological distress. In other words, women who used maladaptive strategies to cope with the conflicting demand from work and family domains are more likely to suffer from poor mental health outcomes. Previous findings showed that avoidant coping, which is considered as a maladaptive style, mediated the association between stressful situations and psychological distress [27, 28]. Therefore, work-family conflict as a potential source of stress leads employees toward using maladaptive coping strategies, which consequently intensify the mental health burden associated with work-family conflict.

This study is able to contribute to the knowledge in coping with work-family conflict and its associated outcomes through several ways. Firstly, helping Malaysian women to identify their salient role and prioritize their domains might lead to lower levels of work-family conflict and reduce its adverse effect on mental health. This goal can be achieved by interventions aimed at increasing knowledge of working women about their valued domain, salient roles, and recognition of early symptoms of work-family conflict, which can prevent or mitigate its level.

Secondly, educating Malaysian working women to adopt and implement coping skills, which may help them to cope with the conflicting demands of work and family effectively, in turn will improve their mental health status. Future interventions targeted at improvement of coping skills and the art of time management may help working women to balance work and family demands. The role of career psychologist would be prominent here to empower employees to become more capable of coping with interroles conflict. The noticeable issue is an adaptation of coping strategies, which are personally oriented and according to each client's specific context. In other words, coping strategies should be according to the need and expectation of each individual. In sum, effective coping strategies need to be developed for controlling the events but not to eliminate the interroles conflict.

There are some limitations to this study which need to be addressed. The first limitation is the use of a cross-sectional design. This design does not allow us to make a causal interpretation. In particular, our results did not support the buffering effect of adaptive coping on the association between work-family conflict and psychological distress, which might be due to lack of measuring long-term effect. However, O'Driscoll et al. [29] revealed that the effect of work-family conflict on psychological distress was stronger at baseline compared to the three-month lag. Therefore, cross-sectional design could be used in the field of work and family conflict research. The second limitation of the current study is the use of self-report data (common method variance bias). Measuring factors, such as coping strategies and perceived level of work-family conflict, which are subjective variables, are fit to use in the self-reported data. However, it would be useful to use multimethod approaches, such as a combination of self-report and observational design, to conduct such studies. The third limitation of this study is the fact that only one member of the family is addressed in this study, which is the working woman. This does impact the study results as a survey of the father and children may provide another side of the story to the assessment. This should be served as an opportunity for future researches on Malaysians, to assess not only the women but also their partner and children.

Despite improvements in developing and implementing family-friendly policies, which help to mitigate the level of work-family conflict, there has been little attention to the role of individual behavioural coping strategies in Malaysia. Malaysian employees may use different combinations of coping strategies for managing stress that is evoked by work-family demand. Therefore, it would be useful to conduct a person-oriented analytical method in order to deeply understand the effect of coping strategies on work-family conflict among Malaysian working women.

## Figures and Tables

**Table 1:** Mean and standard deviation of the behavioural coping strategies.

Strategies	Mean	SD
Religion	4.77	1.42
Active coping	4.11	1.42
Planning	4.09	1.36
Positive reframing	3.98	1.49
Acceptance	3.96	1.53
Seeking instrumental support	3.75	1.57
Self-distraction	3.75	1.53
Seeking emotional support	3.35	1.67
Venting	2.61	1.42
Humor	2.14	1.75
Self-blame	2.04	1.47
Denial	0.99	1.32
Behavioral disengagement	0.99	1.32
Substance use	0.14	0.65

**Table 2:** Descriptive statistics and intercorrelation of variables.

	Mean ± SD	1	2	3	4	5	6	7	8	9
(1) Age	34.7 ± 8.9	1	0.59^**^	0.87^**^	−0.13^**^	−0.01	−0.05	−0.26^**^	−0.12^*^	−0.12^*^
(2) Number of children	1.7 ± 1.5		1	0.54^**^	−0.08	0.05	−0.02	−0.16^**^	−0.11^*^	−0.12^*^
(3) Work experience	10.6 ± 9.3			1	−0.12^*^	−0.08	−0.09	−0.28^**^	−0.09	−0.14^**^
(4) Work hour	42.7 ± 5.2				1	−0.01	−0.04	0.07	0.03	−0.03
(5) WIF	28.5 ± 7.0					1	0.53^**^	−0.10^*^	0.16^**^	0.35^**^
(6) FIW	24.5 ± 6.0						1	−0.01	0.22^**^	0.34^**^
(7) Adaptive	36.5 ± 10.7							1	0.24^**^	−0.04
(8) Maladaptive	4.2 ± 3.7								1	0.40^**^
(9) GHQ	9.8 ± 3.9									1

∗ is significant at *P* < 0.05; ∗∗ is significant at *P* < 0.01; WIF: Work Interference into Family; FIW: Family Interference into Work.

**Table 3:** Linear regression analysis.

Variables	WIF→adaptive→GHQ			WIF→maladaptive→GHQ			FIW→maladaptive→GHQ		
	1	2	3	1	2	3	1	2	3
Step 1: controls									
Work hour	−0.06	−0.05	−0.05	−0.06	−0.05	−0.05	−0.06	−0.04	−0.05
Work experience	−0.12	−0.08	−0.09	−0.12	−0.08	−0.07	−0.12	−0.08	−0.07
Number of children	−0.06	−0.10	−0.10	−0.06	−0.10	−0.07	−0.06	−0.07	−0.05
Education	−0.03	−0.09	−0.09	−0.03	−0.09	−0.07	−0.03	−0.04	−0.03

Step 2: antecedents									
WIF		0.36^**^	0.36^*^		0.36^**^	0.30^**^		—	—
FIW		—	—		—	—		0.33^**^	0.26^**^

Step 3: Mediators									
Adaptive coping			−0.02			—			—
Maladaptive			—			0.33^**^			0.33^**^

*R*	0.02	0.14	0.14	0.02	0.14	0.25	0.02	0.12	0.22

∗ is significant at <0.05; ∗∗ is significant at <0.001. WIF: Work Interference into Family; FIW: Family Interference into Work.
